# Supplementary material for: TGFB2 Gene Methylation in Tumors with Low CD8+ T-Cell Infiltration Drives Positive Prognostic Overall Survival Responses in Pancreatic Ductal Adenocarcinoma
Source: Int J Mol Sci. 2025 Jun 10;26(12):5567. doi: 10.3390/ijms26125567 (PMC12193574; doi:10.3390/ijms26125567)
Supplement: Supplementary file 1 [file ijms-26-05567-s001.zip › ijms-3647117-supplementary.pdf]

Supplementary Files.

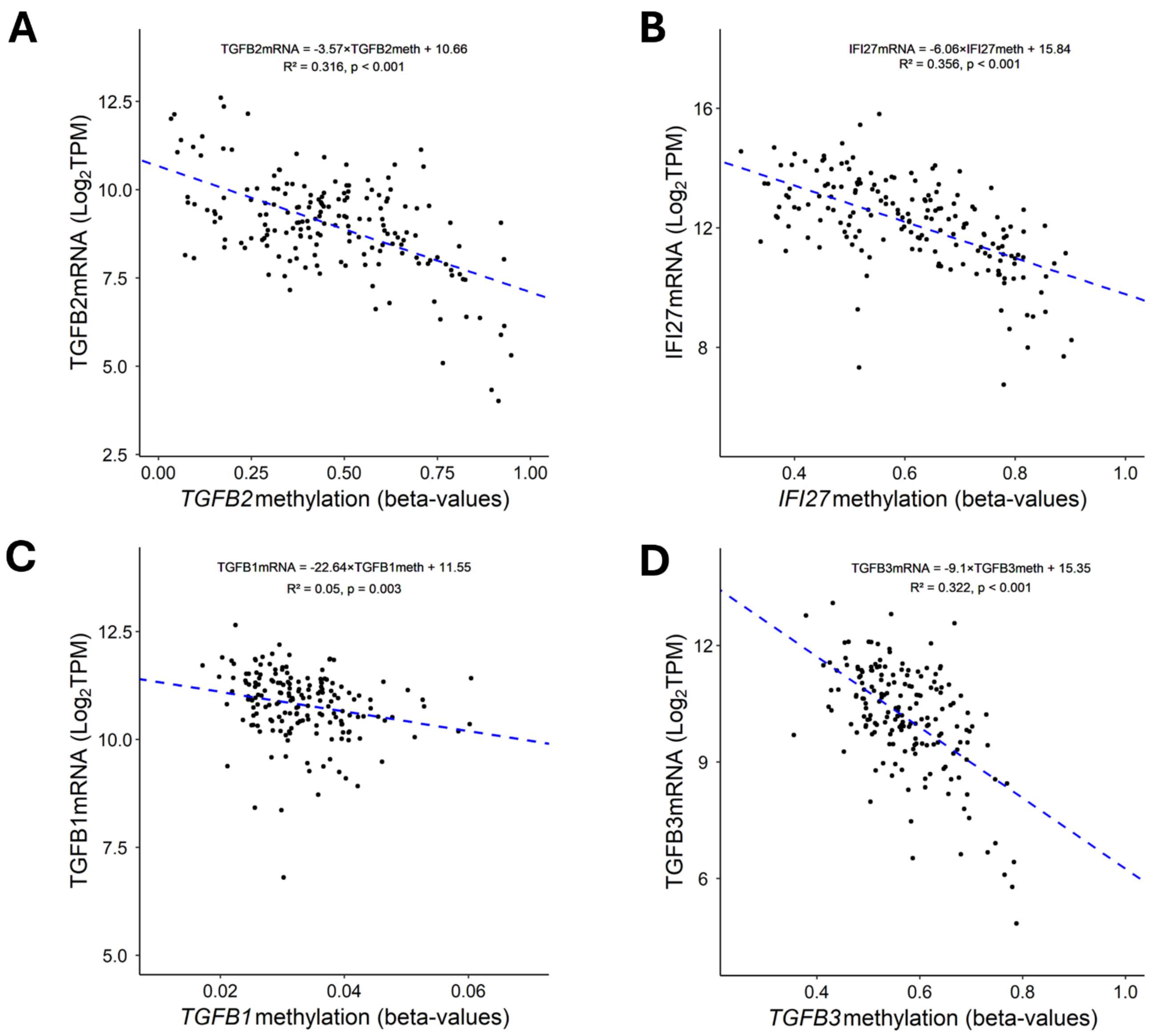

Figure S1. Correlation of *TGFB1/2/3 and IFI27 gene* methylation levels to corresponding mRNA levels in PDAC tumors. Beta-values for *TGFB1/2/3* and *IFI27* gene methylation were correlated with the corresponding mRNA expression values (Log_2 transformed TPM values) for all PDAC patients (N=177 evaluable patients) evaluating TGFB2 (A), IFI27 (B), TGFB1 (C), and TGFB3 (D) relationships.

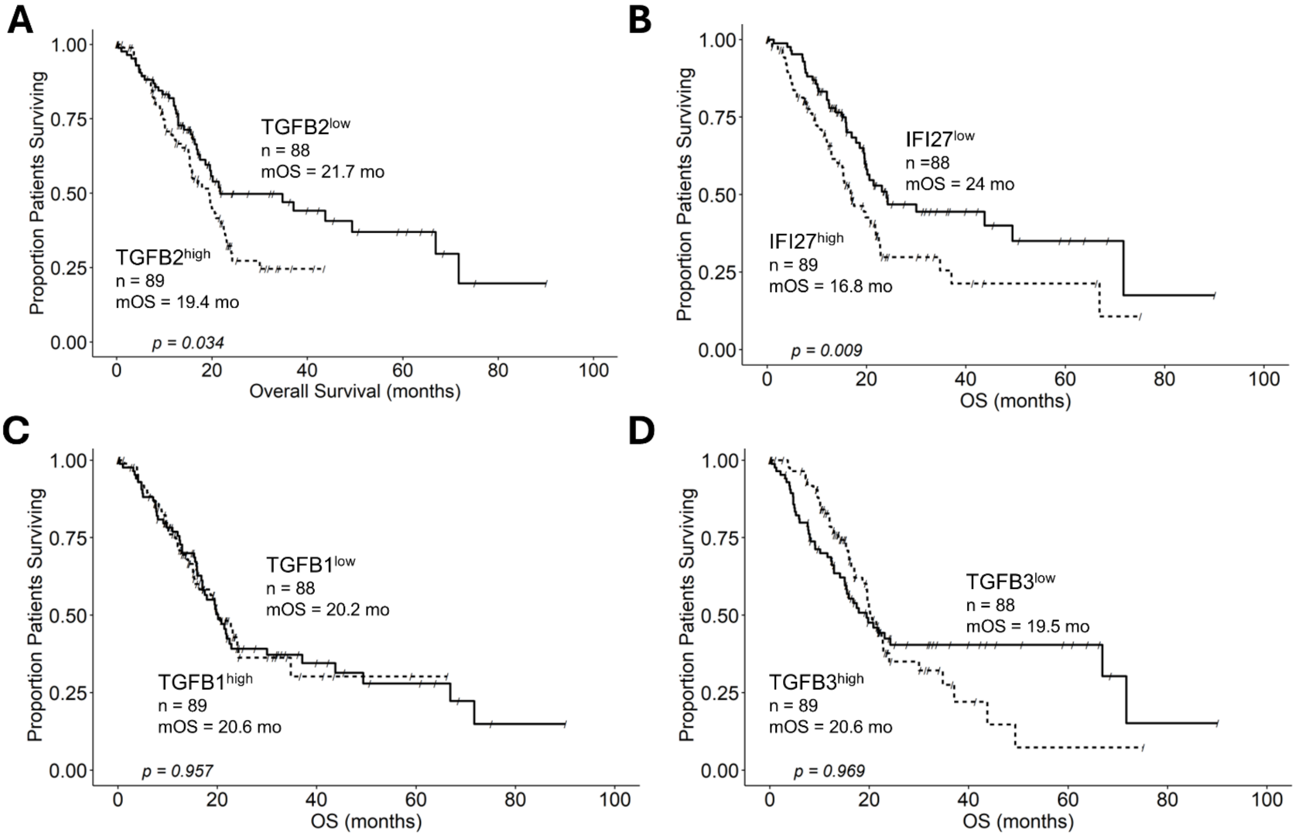

Figure S2. Negative prognostic OS impacts of high levels of TGFB2 and IFI27 mRNA expression in PDAC patients. PDAC patients were correlated to OS outcomes (calculated median OS times; mOS), investigating the impact of mRNA expression of TGFB2 (A), IFI27 (B), TGFB1 (C), and TGFB3 (D) mRNA levels (median cut-off for high and low levels of expression). (**A**) TGFB2^{high} subset of patients (mOS = 19.4; 95% CI = 15.34 - 22.71 months; n = 89; # death events = 51) exhibited a significantly shorter OS outcome than TGFB2^{low} patients (mOS = 21.71; 95% CI = 18.66 - NA months; n = 88; #death events = 41; Log-rank Chi-Square = 4.52, *p* = 0.034). (**B**) IFI27^{high} patients (mOS = 16.8; 95% CI = 15.12 - 22.47 months; N = 89; #death events = 52) exhibited a significantly shorter OS outcome than IFI27^{low} patients (mOS = 24.05; 95% CI = 19.65 - NA months; n = 88; #Events = 40; Log-rank Chi-Square = 6.77, *p* = 0.009). (**C**) TGFB1^{high} patients (mOS = 20.6; 95% CI = 15.44 - NA months; n = 89; #death events = 42) exhibited very similar OS outcome compared to TGFB1^{low} patients (mOS = 20.17; 95% CI = 17 - 37.12 months; N = 88; #death events = 50; Log-rank Chi-Square = 0, *p* = 0.957). (**D**) TGFB3^{high} patients (mOS = 20.6; 95% CI = 18.66 - 30.02 months; N = 89; #death events = 45) exhibited similar median OS outcome to TGFB3^{low} patients (mOS = 19.48; 95% CI = 15.12 - NA months; N = 88; #death events = 47; Log-rank Chi-Square = 0, P-value = 0.969). The correlations for *TGFB2* and *IFI27* gene methylation and mRNA products were prognostically correlated with OS outcomes, comparing high versus low expression. This was not observed for TGFB1 and TGFB3 mRNA correlation with OS outcomes.

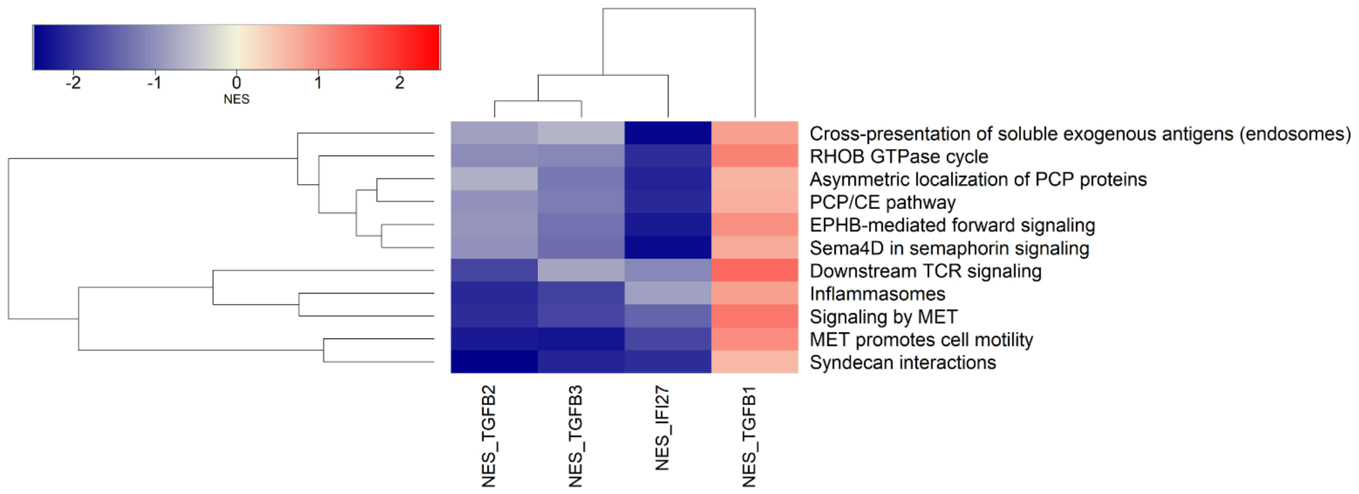

Figure S3. Identification of Reactome pathways negatively correlated to *TGFB2/3 and IFI27 gene* methylation. Beta-values for *TGFB1/2/3* and *IFI27* gene methylation were correlated using Spearman ranks with mRNA expression levels of 14861 genes for all PDAC patients (N=177 evaluable patients) across 1286 Reactome pathways. The cluster figure depicts the Normalized Enrichment Scores (NES) scores for *TGFB1/2/3 and IFI27* methylation (NES_TGFB1, NES_TGFB2, NES_TGFB3, NES_IFI27). We subsequently applied a p-value filter whereby significant enrichments were observed (NES $p<0.0001$) for correlations for *TGFB1/2/3* or *IFI27* gene methylations that identified 222 pathway descriptions. Next, we identified 11 pathways negatively correlated with *TGFB2/3* and *IFI27* gene methylations and positively correlated with *TGFB1* gene methylation. 351 genes represented these 11 pathways.

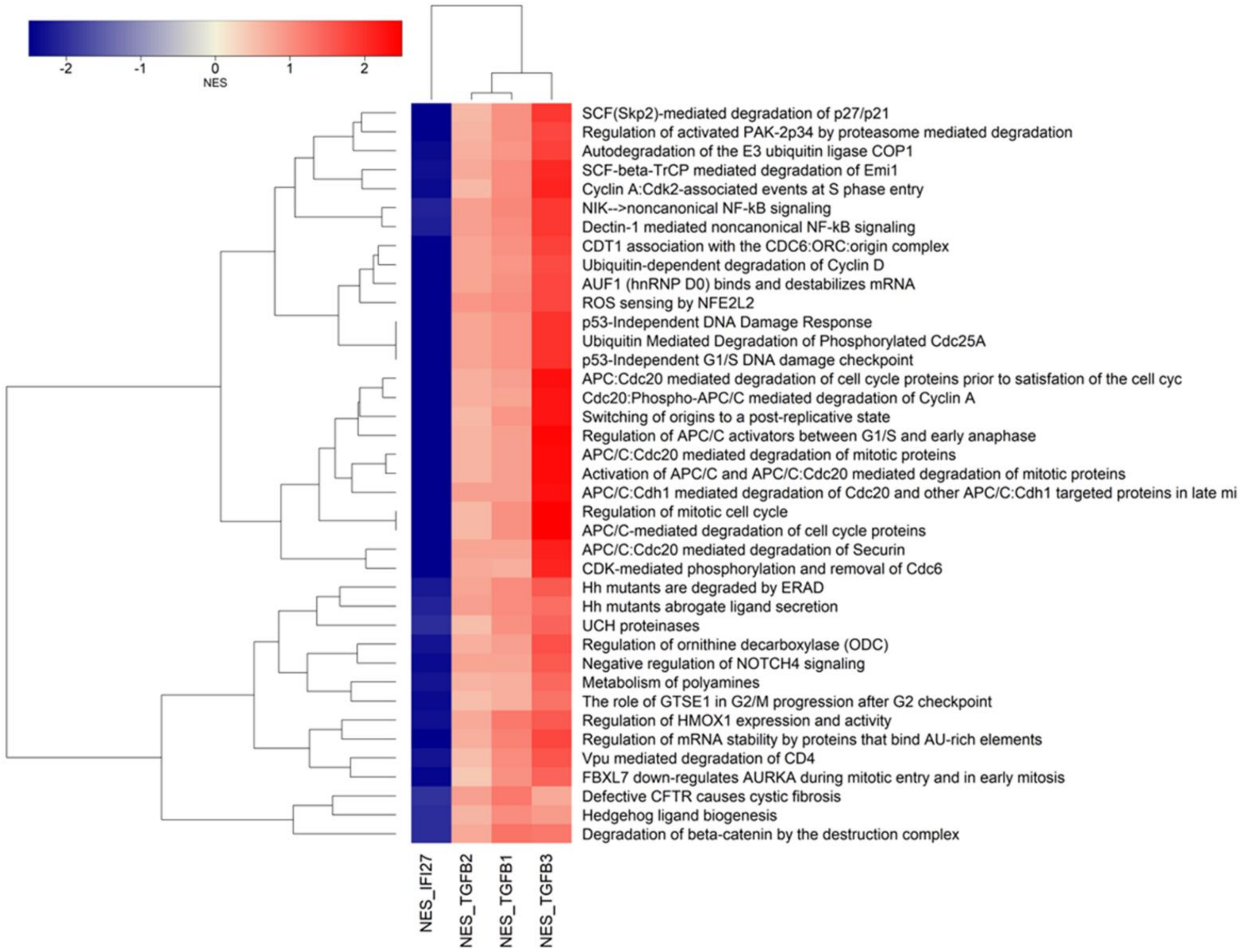

Figure S4. Identification of Reactome pathways negatively correlated to *IFI27 gene* methylation. Beta-values for *TGFB1*/*2*/*3* and *IFI27* gene methylation were correlated using Spearman ranks with mRNA expression levels of 14861 genes for all PDAC patients (N=177 evaluable patients) across 1286 Reactome pathways. The cluster figure depicts the Normalized Enrichment Scores (NES) for *TGFB1*/*2*/*3* and *IFI27* methylation (NES_TGFB1, NES_TGFB2, NES_TGFB3, NES_IFI27). We subsequently applied a P-value filter whereby significant enrichments were observed (NES $p<0.0001$) for correlations for *TGFB1*/*2*/*3* or *IFI27* gene methylations that identified 222 pathway descriptions. Next, we identified 39 pathways negatively correlated with *IFI27* gene methylations.

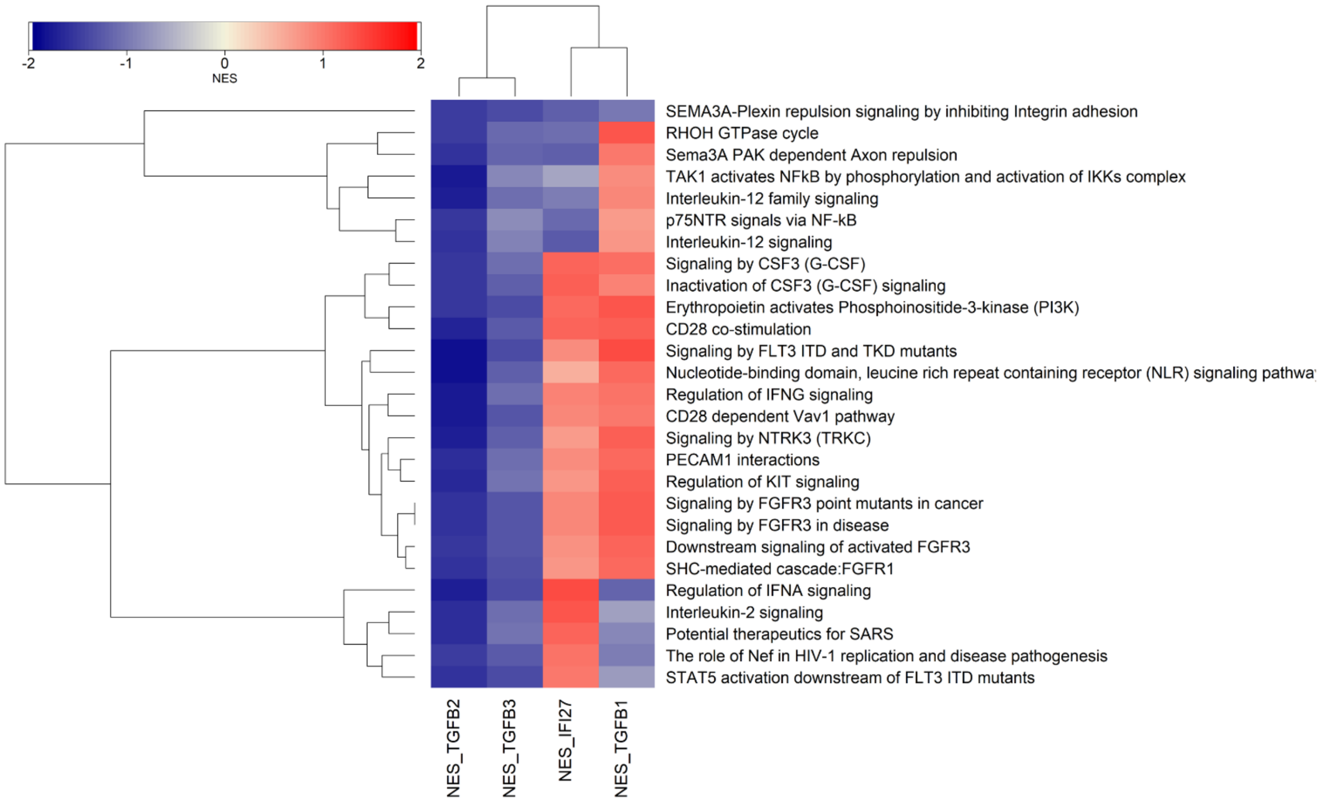

Figure S5. Identification of Reactome pathways negatively correlated to *TGFB2* gene methylation. Beta-values for *TGFB1/2/3* and *IFI27* gene methylation were correlated using Spearman ranks with mRNA expression levels of 14861 genes for all PDAC patients (N=177 evaluable patients) across 1286 Reactome pathways. The cluster figure depicts the Normalized Enrichment Scores (NES) for *TGFB1/2/3 and IFI27* methylation (NES_TGFB1, NES_TGFB2, NES_TGFB3, NES_IFI27). We subsequently applied a P-value filter whereby significant enrichments were observed (NES p<0.0001) for correlations for *TGFB1/2/3* or *IFI27* gene methylations that identified 222 pathway descriptions. Next, we identified 27 pathways using the filter: pval *TGFB2* methylation < 0.05 & NES *TGFB2* methylation < 0 & pval *TGFB1* methylation > 0.1 & pval *TGFB3* methylation > 0.1 & pval *IFI27* methylation > 0.1. This filter identified 358 genes that represent these 27 pathways.

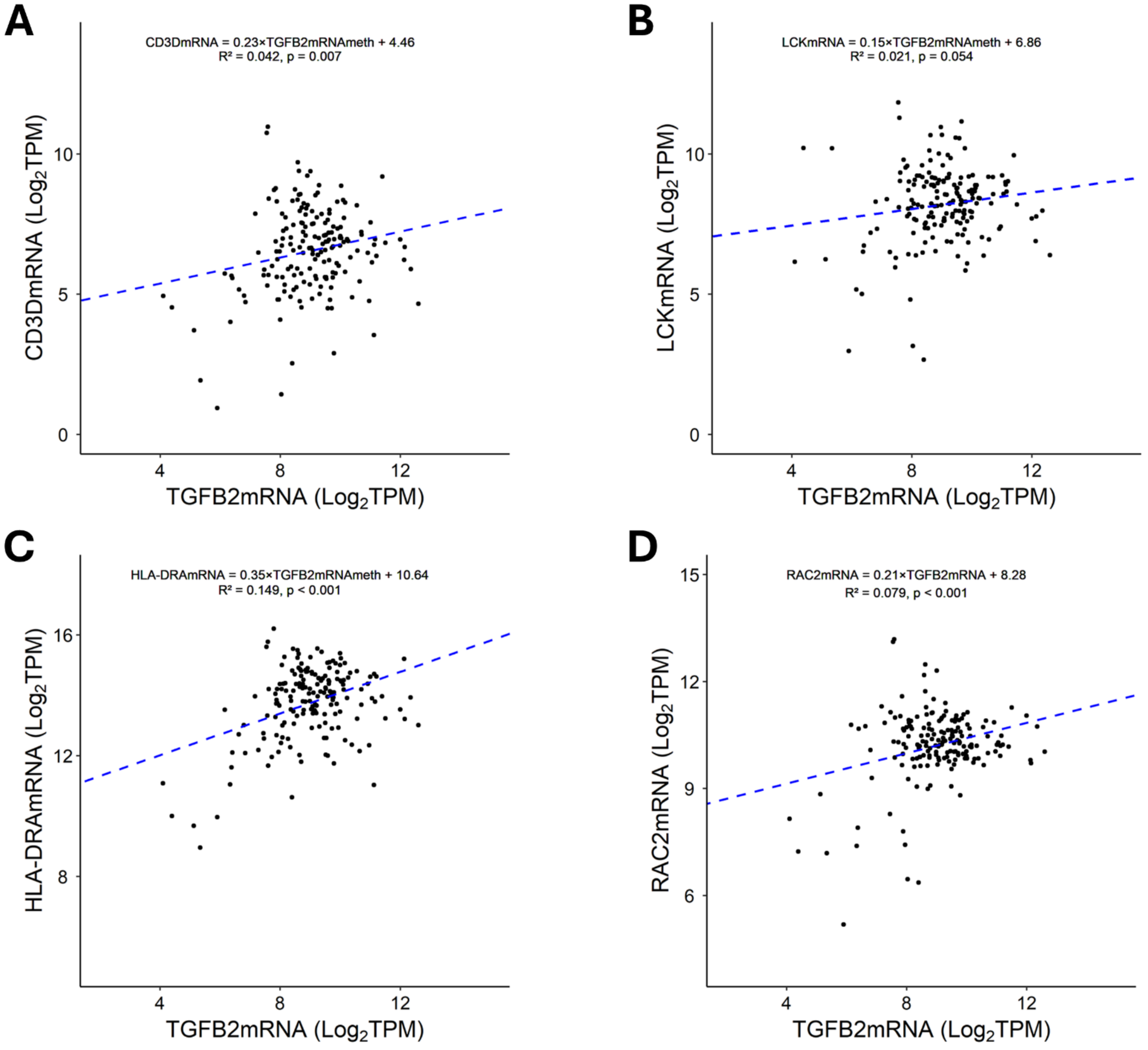

Figure S6. Correlation of TGFB2 mRNA to the marker genes. Expression of TGFB2 mRNA was correlated to the four marker genes. Log$_2$ transformed TPM values for TGFB2 mRNA were correlated to CD3D (A), LCK (B), HLA-DRA (C) and RAC2 (D) mRNA for evaluable PDAC patients (N=177). Weak correlations were observed for CD3D ($R^2 = 0.042$, $p = 0.007$), LCK mRNA ($R^2 = 0.021$, $p = 0.054$), and RAC2 ($R^2 = 0.079$, $p < 0.001$) with TGFB2 mRNA. Correlation of HLA-DRA with TGFB2 mRNA explained greater than 10% of the variation ($R^2 = 0.149$, $p < 0.001$).

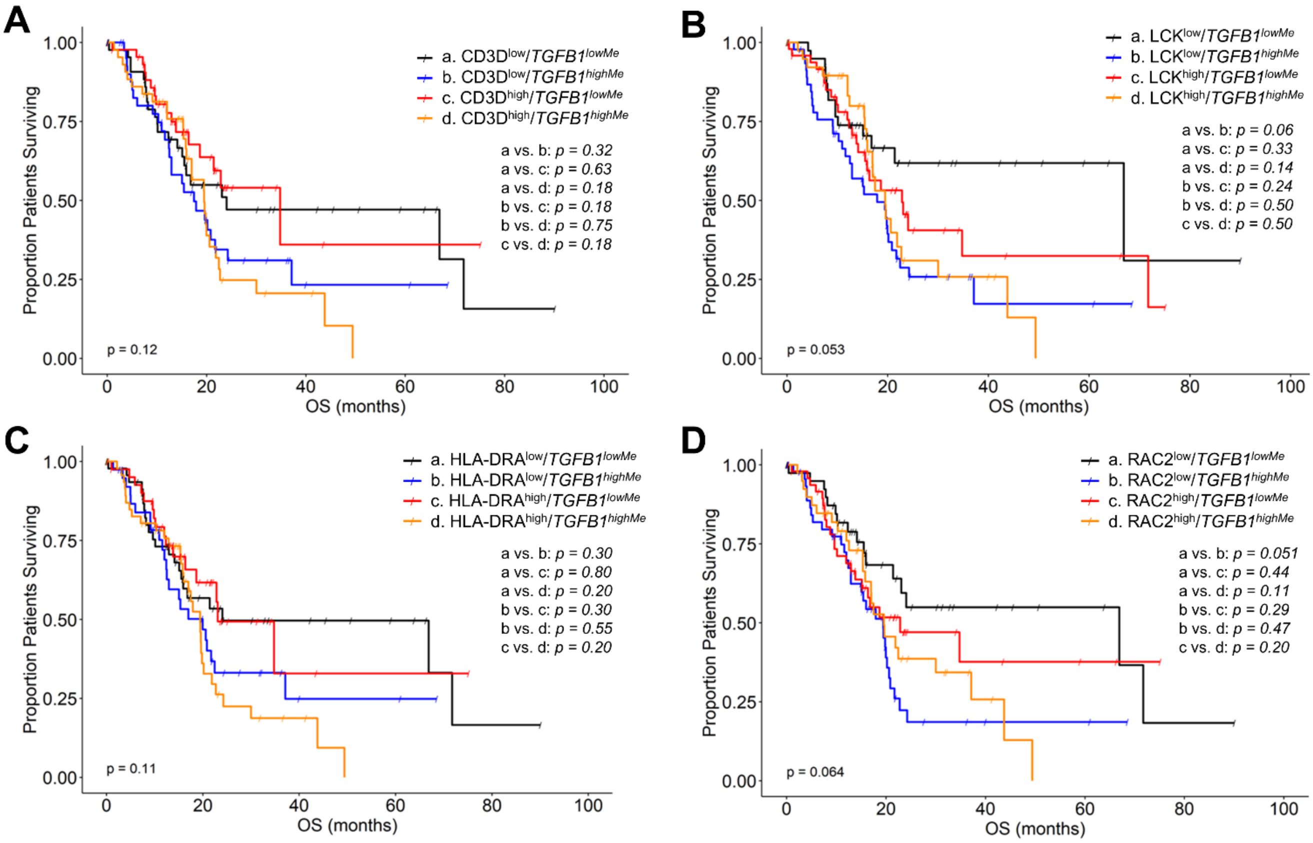

Figure S7 Prognostic OS impacts of *TGFB1* gene methylation in combination with marker gene expression levels in PDAC patients. PDAC patients were correlated to OS outcomes, investigating the impact of methylation beta-values (median cut-off for high methylation levels; superscripted "highMe" compared to low methylation; "lowMe" for *TGFB1* methylation), further stratified into four groups based on gene expression levels of CD3D (A), LCK (B), HLA-DRA (C), and RAC2 (D) mRNA expression levels (median cut-off for low and high values) in these patients. The Kaplan-Meier plots show four stratified curves for each of the marker genes. Six pairwise comparisons were performed between the four groups of patients (p-value adjusted using the BH correction). [**A**] The mOS for 44 patients from the group CD3D^{low}/*TGFB1*^{lowMe} was 24.1 months (95% CI: 15.5 – NA, death events = 22). For 44 patients in the group CD3D^{low}/*TGFB1*^{highMe}, the mOS was 17.5 months (95% CI: 12.9 – 37.1, death events = 26). The mOS for 45 patients in the group CD3D^{high}/*TGFB1*^{lowMe} was 34.8 months (95% CI: 18.7 – NA, death events = 16), while for 44 patients in the group CD3D^{high}/*TGFB1*^{highMe}, it was 19.5 months (95% CI: 16 – 22.7, death events = 28).[**B**] The mOS for 40 patients in the group LCK^{low}/*TGFB1*^{lowMe} was 66.9 months (95% CI: 21.4 - NA, death events = 14). In the LCK^{low}/*TGFB1*^{highMe} group, the mOS for 48 patients was 17.9 months (95% CI: 12.5 - 21.7, death events = 32). For 49 patients in the LCK^{high}/*TGFB1*^{lowMe} group, the mOS was 22.8 months (95% CI: 15.5 - NA, death events = 24). Lastly, the mOS for 40 patients in the LCK^{high}/*TGFB1*^{highMe} group was 19.5 months (95% CI: 17.0 - NA, death events = 22). At low levels of LCK mRNA expression, increasing *TGFB1* gene methylation resulted in worse OS outcomes but did not achieve statistical significance; OS outcomes for patient groupings were as follows: LCK^{low}/*TGFB1*^{lowMe} (N = 40, Median OS = 66.9 months) and LCK^{low}/*TGFB1*^{highMe} (N = 48, Median OS = 17.9 months, OS difference = −48.99, p = 0.060). [**C**] The mOS for 47 patients from the HLA-DRA^{low}/*TGFB1*^{lowMe} group was 24.1 months (95% CI: 15.54 – NA, death events = 22). For 41 patients in the group HLA-DRA^{low}/*TGFB1*^{highMe}, the mOS was 20 months (95% CI: 12.5 - NA, death events = 23). In the group HLA-DRA^{high}/*TGFB1*^{lowMe}, 42 patients had a mOS of 23.1 months (95% CI: 18.7 - NA, death events = 16). Finally, for 47 patients in the group HLA-DRA^{high}/*TGFB1*^{highMe}, the mOS was 19.4 months (95% CI: 16 – 22.7175593, death events = 31). [**D**] The mOS for 40 patients from the group RAC2^{low}/TGFB1^{lowMe} was 66.9 months (95% CI: 21.4 – NA, death events = 16). In comparison, the mOS for 48 patients from the group RAC2^{low}/TGFB1^{highMe} was 19.4 months (95% CI: 12.9 – 20.8, death events = 30). For 49 patients in the group RAC2^{high}/TGFB1^{lowMe}, the mOS was 22.8 months (95% CI: 15.1 – NA, death events = 22), while for 40 patients in the group RAC2^{high}/TGFB1^{highMe}, the mOS was 19.5943058 months (95% CI: 15.8135253 – NA, death events = 24). There was borderline significant difference in OS outcomes for comparing RAC2^{low}/TGFB1^{lowMe} (N = 40, Median OS = 66.9 months) versus RAC2^{low}/TGFB1^{highMe} (N = 48, Median OS = 19.4 months, OS difference = −47.44, p = 0.051) groups of patients.

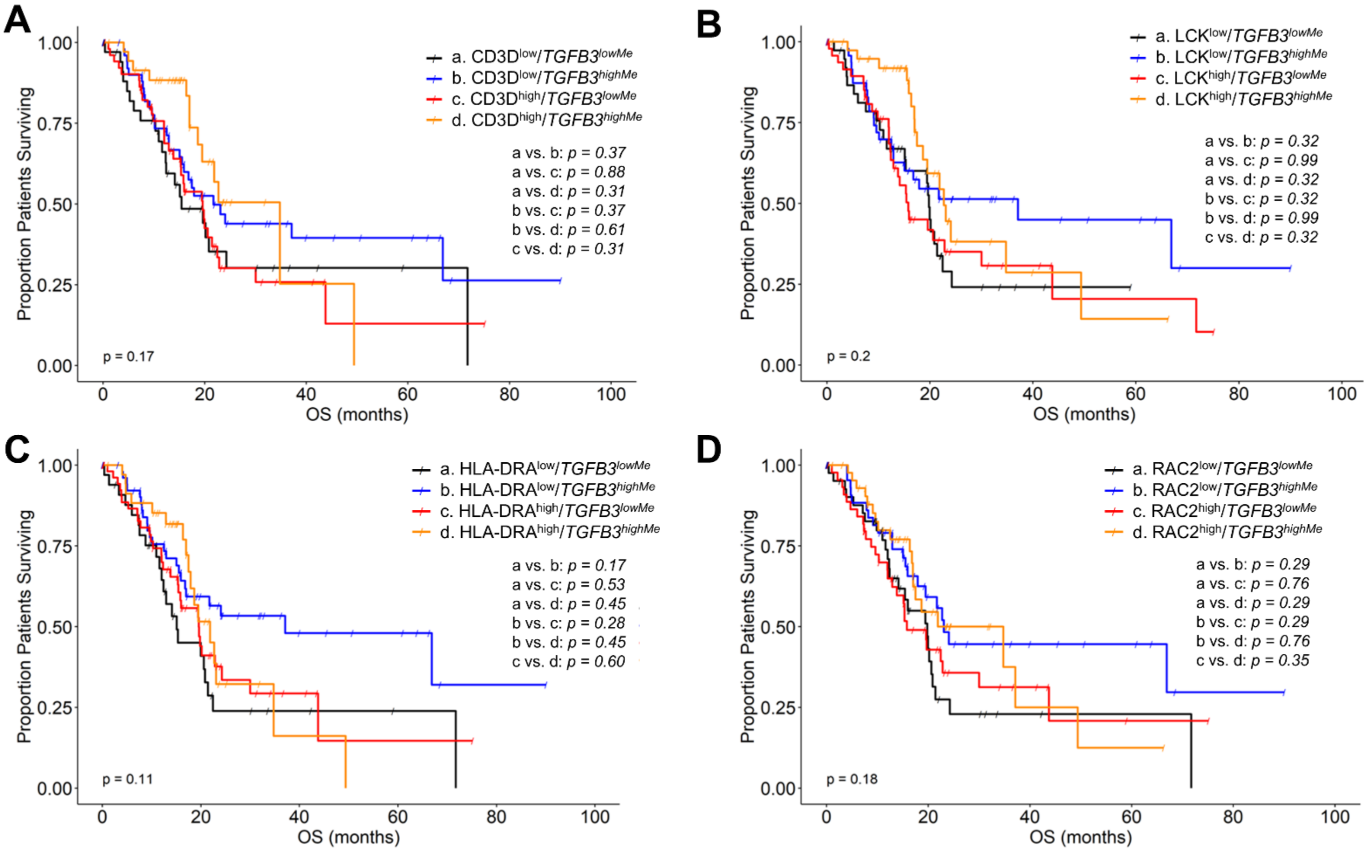

Figure S8 Prognostic OS impacts of *TGFB3* gene methylation in combination with marker gene expression levels in PDAC patients. PDAC patients were correlated to OS outcomes investigating the impact methylation beta values (median cut-off for high methylation levels; superscripted "highMe" compared to low methylation; "lowMe" for *TGFB3* methylation), further stratified according into four groups based on gene expression levels of CD3D (A), LCK (B), HLA-DRA (C), and RAC2 (D) mRNA expression levels (median cut-off for low and high values) in these patients. The Kaplan-Meier plots show four stratified curves for each of the marker genes. Six pairwise comparisons were performed between the four groups of patients (p-value adjusted using the BH correction). [**A**] The mOS for 35 patients from the CD3D^{low}/*TGFB3*^{lowMe} group was 15.4 months (95% CI: 12.3 – NA, death events = 21). For the CD3D^{low}/*TGFB3*^{highMe} group, consisting of 53 patients, the mOS was 21.7 months (95% CI: 15.5 – NA, death events = 27). The mOS for 53 patients from the group CD3D^{high}/*TGFB3*^{lowMe} was 19.6 months (95% CI: 15.3 – 22.8, death events = 31). Finally, for the group CD3D^{high}/*TGFB3*^{highMe}, which included 36 patients, the mOS reported was 34.8 months (95% CI: 19.5 – NA, death events = 13). [**B**] For 39 patients in the LCK^{low}/*TGFB3*^{lowMe} group, the mOS was found to be 19.8 months (95% CI: 15.1 – 24.3, with 23 death events). The mOS for 49 patients in the LCK^{low}/*TGFB3*^{highMe} group was 37.1 months (95% CI: 12.9 – NA, with 23 death events). Additionally, the mOS for another 49 patients in the LCK^{high}/*TGFB3*^{lowMe} was 15.8 months (95% CI: 12.9 – NA, with 29 death events). Finally, for 40 patients in the LCK^{high}/*TGFB3*^{highMe} group, the mOS was 22.7 months (95% CI: 18.7 – NA, with 17 death events). [**C**] The mOS for 34 patients in the HLA-DRA^{low}/*TGFB3*^{lowMe} group was 15.1 (95% CI: 12.0 – 22.5, death events = 22) months. For 54 patients in the HLA-DRA^{low}/*TGFB3*^{highMe} group, the mOS was 37.1 (95% CI: 16.8 – NA, death events = 23) months. The mOS for 54 patients in the HLA-DRA^{high}/*TGFB3*^{lowMe} group was 19.6 (95% CI: 15.44 – NA, death events = 30) months, while for 35 patients in the HLA-DRA^{high}/*TGFB3*^{highMe} group, it was 21.9 (95% CI: 17.9 – NA, death events = 17) months.[**D**] The mOS for 43 patients in the RAC2^{low}/*TGFB3*^{lowMe} group was 19.8 (95% CI: 14.1 – 24.3, death events = 25) months. For the RAC2^{low}/*TGFB3*^{highMe} group, 45 patients had a mOS of 23.1 (95% CI: 17.9 – NA, death events = 21) months. In the RAC2^{high}/*TGFB3*^{lowMe} group, the mOS for 45 patients was 15.8 (95% CI: 12.9 – NA, death events = 27) months. Lastly, 44 patients in the RAC2^{high}/*TGFB3*^{highMe} group experienced a mOS of 21.9 (95% CI: 17.0 – NA, death events = 19) months.

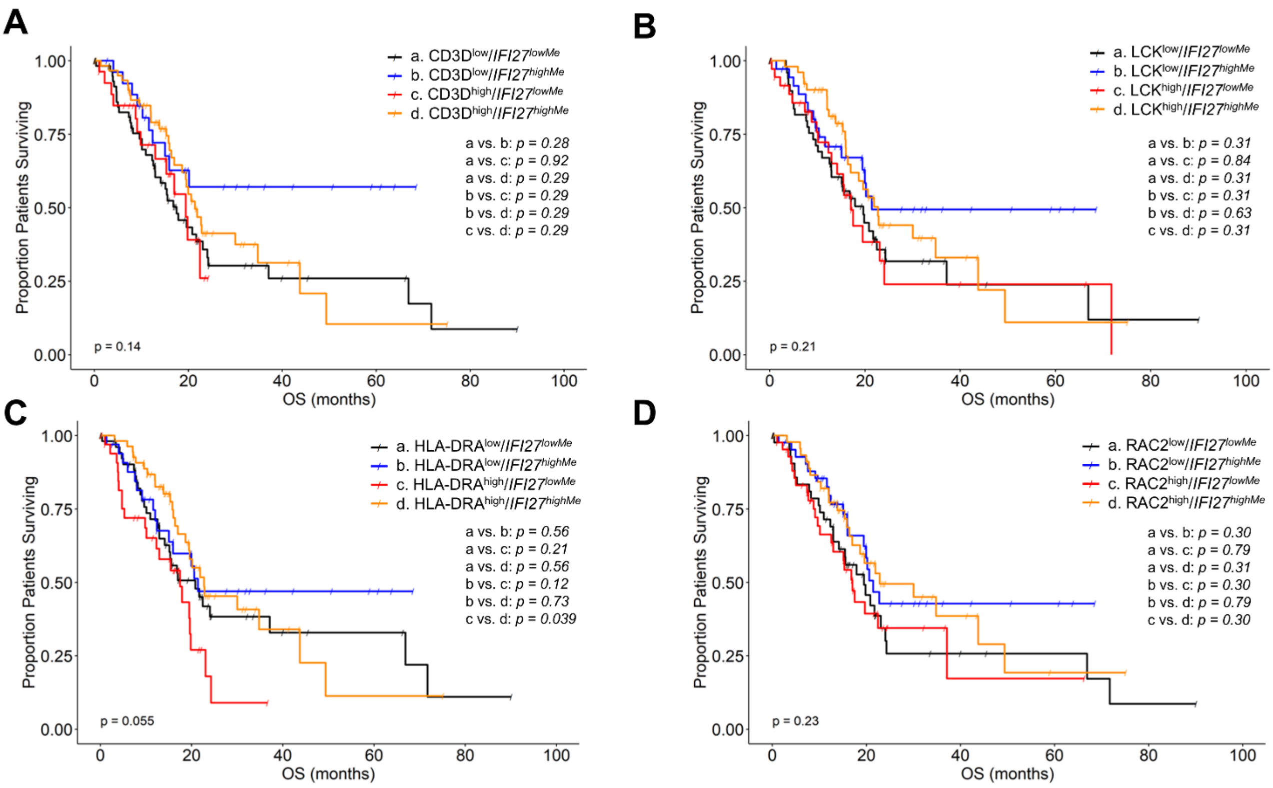

Figure S9 Prognostic OS impacts of *IFI27* gene methylation in combination with marker gene expression levels in PDAC patients. PDAC patients were correlated to OS outcomes, investigating the impact of methylation beta-values (median cut-off for high methylation levels; superscripted "highMe" compared to low methylation; "lowMe" for *IFI27* methylation), further stratified into four groups based on gene expression levels of CD3D (A), LCK (B), HLA-DRA (C), and RAC2 (D) mRNA expression levels (median cut-off for low and high values) in these patients. The Kaplan-Meier plots show four stratified curves for each of the marker genes. Six pairwise comparisons were performed between the four groups of patients (p-value adjusted using the BH correction). [**A**] The mOS for 61 patients in the group CD3D^{low}/*IFI27*^{lowMe} was 17.5 (95% CI: 12.9 – 24.3, death events = 38) months. For 27 patients in the CD3D^{low}/*IFI27*^{highMe} group, the mOS was NA (95% CI: 15.9 – NA, death events = 10) months. In the CD3D^{high}/*IFI27*^{lowMe} group, the mOS for 27 patients was 19.5 (95% CI: 12.9 – NA, death events = 13) months, while for 62 patients in the CD3D^{high}/*IFI27*^{highMe} group, the mOS was 21.4 (95% CI: 18.7 – NA, death events = 31) months. [**B**] The mOS for 51 patients in the LCK^{low}/*IFI27*^{lowMe} group was 19.6 months (95% CI: 12.9 – NA, death events = 31). In the LCK^{low}/*IFI27*^{highMe} group, 37 patients had a mOS of 21.4 months (95% CI: 19.4 – NA, death events = 15). For 37 patients in the LCK^{high}/*IFI27*^{lowMe} group, the mOS was 17.0 months (95% CI: 14.1 – NA, death events = 20). Lastly, the mOS for 52 patients in the LCK^{high}/*IFI27*^{highMe} group was 22.7 months (95% CI: 17.0 – NA, death events = 26). [**C**] The mOS for 54 patients in the HLA-DRA^{low}/*IFI27*^{lowMe} group was 20.8 (95% CI: 15.1 – NA, death events = 30) months. In the HLA-DRA^{low}/*IFI27*^{highMe} group of 34 patients, the mOS was 21.4 (95% CI: 15.1 – NA, death events = 15) months. Another 34 patients in the HLA-DRA^{high}/*IFI27*^{lowMe} group had a mOS of 17.5 (95% CI: 12.3 – NA, death events = 21) months. Lastly, for 55 patients in the HLA-DRA^{high}/*IFI27*^{highMe} group, the mOS was 22.7 (95% CI: 19.45 – NA, death events = 26) months. There was a significant difference in OS outcomes comparing HLA-DRA^{high}/*IFI27*^{lowMe} (N = 34, Median OS = 17.5 months) versus HLA-DRA^{high}/IFI27^{highMe} (N = 55, Median OS = 22.7 months, OS difference = 5.2, p = 0.039) groups of patients. [**D**] The mOS for 45 patients in the RAC2^{low}/*IFI27*^{lowMe} group was 19.5 months (95% CI: 14.1 – 24.3, death events = 28). The median for 43 patients in the RAC2^{low}/*IFI27*^{highMe} group was 21.4 months (95% CI: 19.4 – NA, death events = 18). In the RAC2^{high}/*IFI27*^{lowMe} group with 43 patients, the mOS was 17.0 months (95% CI: 12.5 – NA, death events = 23). Lastly, the mOS for 46 patients in the RAC2^{high}/*IFI27*^{highMe} group was 22.8 months (95% CI: 17.0 – NA, death events = 23).

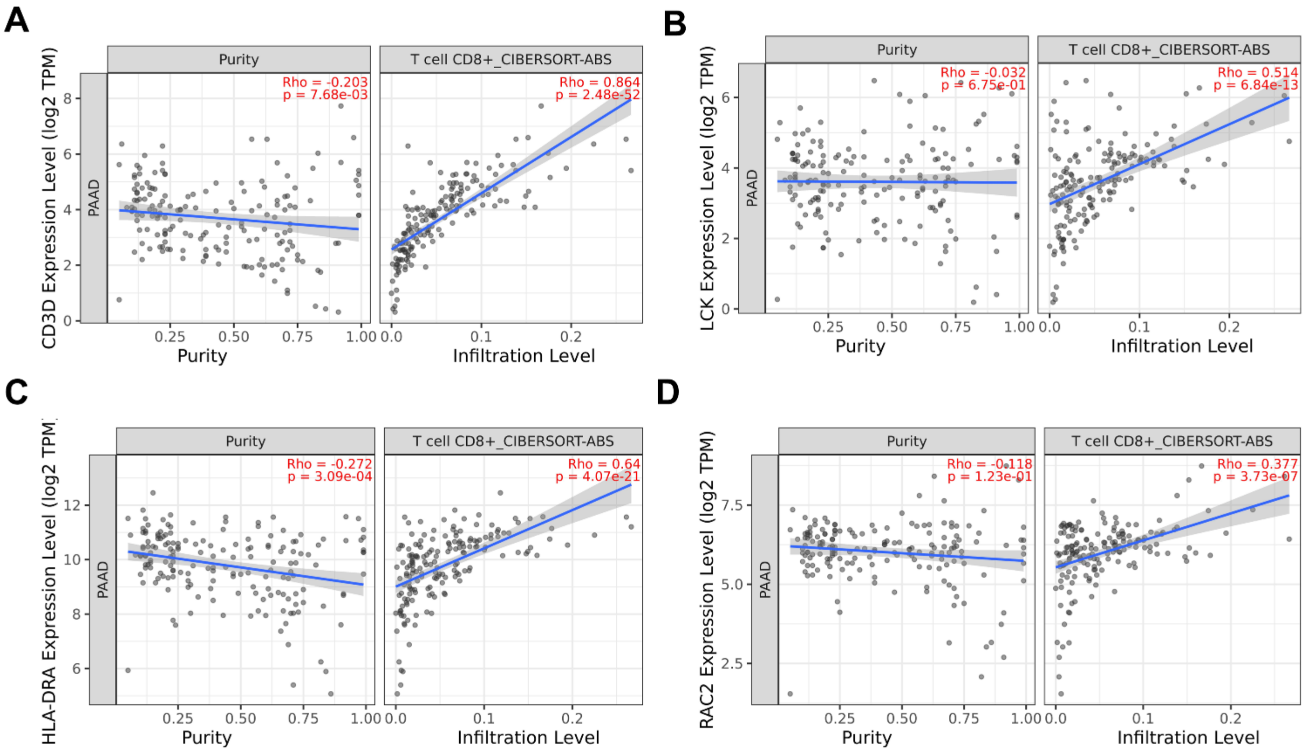

Figure S10 Marker gene mRNA expression levels correlate positively with CD8+ T-cell infiltration into PDAC tumors. We estimated the correlation of CD3D (A), LCK (B), HLA-DRA (C), and RAC2 (D) and CD8+ T-cells infiltration in PDAC tumors using the algorithms provided in the TIMER2.0 (http://timer.cistrome.org/) web tool compiled for the Cancer Genome Atlas (TCGA). Depicted are the purity-adjusted Spearman's rho correlations utilizing CIBERSORT-ABS immune deconvolution methods to estimate infiltration of CD8+ T-cells.

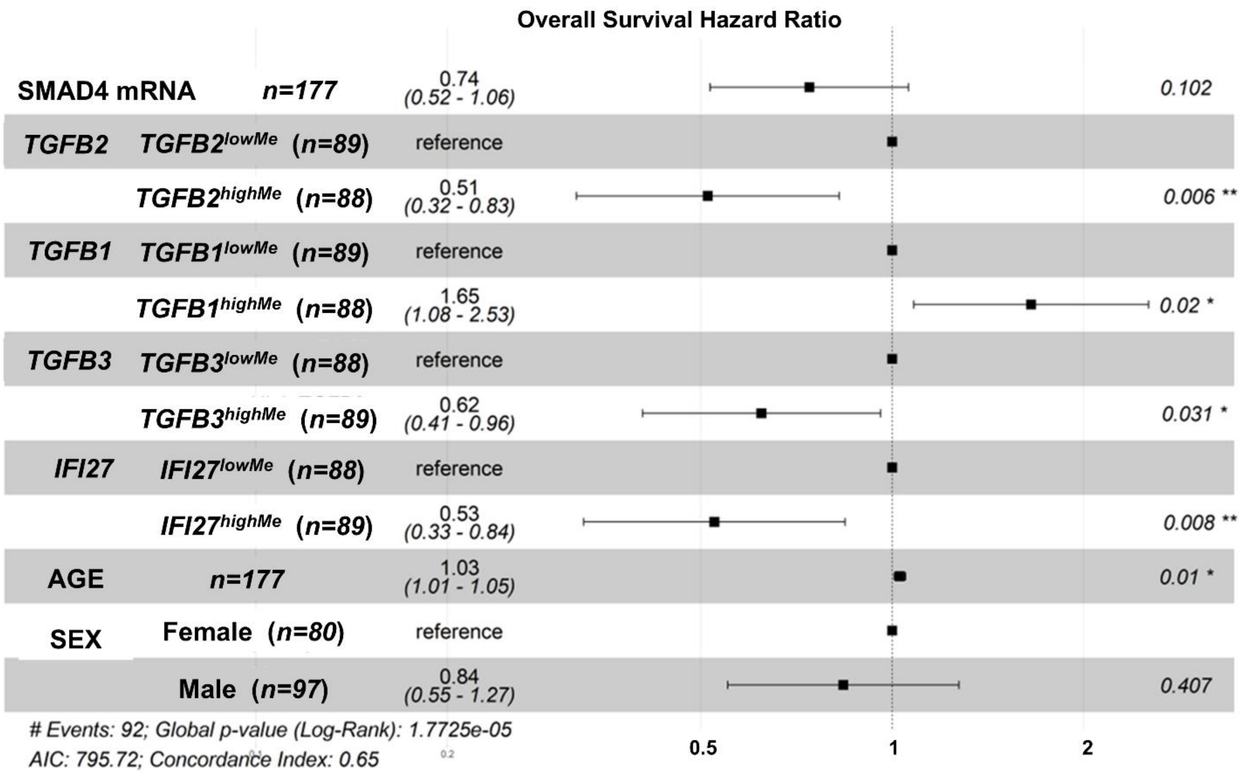

Figure S11. **Independent impacts of SMAD4 and gene methylations, controlling for age and sex, using the Cox proportional hazards model to assess OS.** We used the multivariate model further to substantiate the impact of SMAD4 and gene methylations on OS as independent variables, considering their correlation with each other and other variables, including age and sex. The Forest plot depicts the hazard ratios (HR) for each variable tested with 95% confidence intervals.

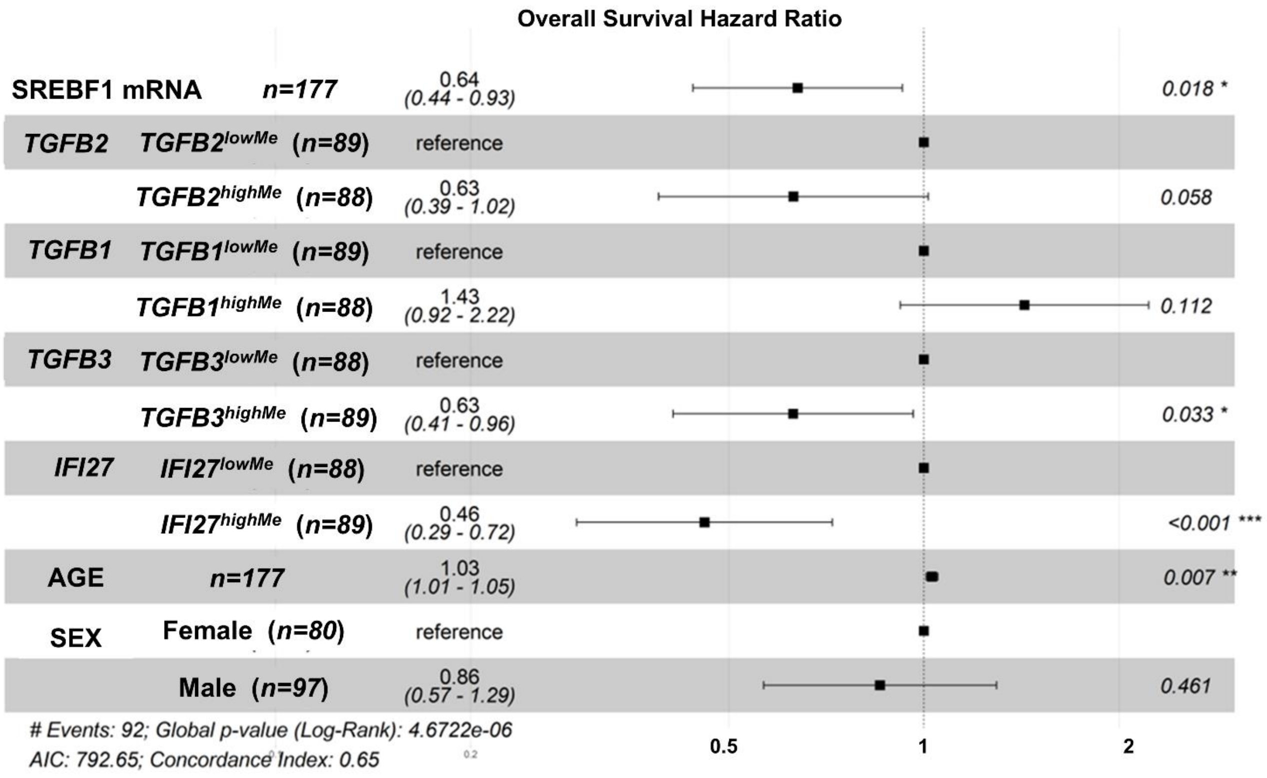

Figure S12. **Independent impacts of SREBF1 and gene methylations, controlling for age and sex, using the Cox proportional hazards model to assess OS.** We used the multivariate model further to substantiate the impact of SREBF1 and gene methylations on OS as independent variables, considering their correlation with each other and other variables, including age and sex. The Forest plot depicts the hazard ratios (HR) for each variable tested with 95% confidence intervals.

Table S1 Reactome pathways negatively correlated with *TGFB2/3* and *IFI27* gene methylations and positively correlated with *TGFB1* gene methylation

Reactome pathway	NES score *TGFB2* methylation	NES score *TGFB3* methylation	NES score IFI27 methylation	NES score *TGFB1* methylation
Syndecan interactions	−2.481	−2.142	−2.021	0.581
MET promotes cell motility	−2.231	−2.295	−1.775	1.048
Signaling by MET	−2.040	−1.751	−1.463	1.238
Inflammasomes	−2.042	−1.809	−0.832	0.860
Downstream TCR signaling	−1.779	−0.801	−1.120	1.394
Sema4D in semaphorin signaling	−0.987	−1.367	−2.369	0.742
EPHB−mediated forward signaling	−0.929	−1.313	−2.222	0.974
PCP/CE pathway	−0.977	−1.188	−2.057	0.667
Asymmetric localization of PCP proteins	−0.681	−1.266	−2.118	0.649
RHOB GTPase cycle	−1.025	−1.103	−2.011	1.138
Cross−presentation of soluble exogenous antigens (endosomes)	−0.823	−0.653	−2.434	0.855

Table S2 *IFI27* gene methylation−specific Reactome pathways

Reactome pathway	NES score *IFI27* methylation	NES score *TGFB2* methylation	NES score *TGFB1* methylation	NES score *TGFB3* methylation
Degradation of beta−catenin by the destruction complex	−2.017	0.754	1.325	1.233
Hedgehog ligand biogenesis	−2.040	0.618	1.070	0.878
Defective CFTR causes cystic fibrosis	−1.983	0.833	1.235	0.764
FBXL7 down−regulates AURKA during mitotic entry and in early mitosis	−2.416	0.458	0.996	1.440
Vpu mediated degradation of CD4	−2.284	0.529	1.042	1.587
Regulation of mRNA stability by proteins that bind AU−rich elements	−2.485	0.670	1.163	1.735
Regulation of HMOX1 expression and activity	−2.347	0.721	1.274	1.559
The role of GTSE1 in G2/M progression after G2 checkpoint	−2.393	0.514	0.687	1.318
Metabolism of polyamines	−2.255	0.646	0.697	1.410
Negative regulation of NOTCH4 signaling	−2.386	0.816	0.817	1.570
Regulation of ornithine decarboxylase (ODC)	−2.280	0.690	0.850	1.641
UCH proteinases	−2.031	0.532	1.003	1.434
Hh mutants abrogate ligand secretion	−2.127	0.826	1.037	1.332
Hh mutants are degraded by ERAD	−2.225	0.816	1.033	1.578
CDK−mediated phosphorylation and removal of Cdc6	−2.500	0.734	0.670	2.097
APC/C:Cdc20 mediated degradation of Securin	−2.500	0.796	0.767	2.176
APC/C−mediated degradation of cell cycle proteins	−2.500	0.585	1.008	2.496
Regulation of mitotic cell cycle	−2.500	0.585	1.008	2.496
APC/C:Cdh1 mediated degradation of Cdc20 and other APC/C:Cdh1 targeted proteins in late mitosis/early G1	−2.500	0.843	0.844	2.342
Activation of APC/C and APC/C:Cdc20 mediated degradation of mitotic proteins	−2.500	0.650	0.820	2.390
APC/C:Cdc20 mediated degradation of mitotic proteins	−2.500	0.654	0.826	2.360
Regulation of APC/C activators between G1/S and early anaphase	−2.457	0.628	0.853	2.400
Switching of origins to a post−replicative state	−2.500	0.582	0.928	2.291
Cdc20:Phospho−APC/C mediated degradation of Cyclin A	−2.500	0.693	0.802	2.265
APC:Cdc20 mediated degradation of cell cycle proteins prior to satisfation of the cell cycle checkpoint	−2.500	0.684	0.832	2.300
p53−Independent G1/S DNA damage checkpoint	−2.500	0.802	0.923	1.969
Ubiquitin Mediated Degradation of Phosphorylated Cdc25A	−2.500	0.802	0.923	1.969

p53−Independent DNA Damage Response	−2.500	0.802	0.923	1.969
ROS sensing by NFE2L2	−2.500	0.927	1.028	1.752
AUF1 (hnRNP D0) binds and destabilizes mRNA	−2.500	0.816	1.002	1.738
Ubiquitin−dependent degradation of Cyclin D	−2.500	0.802	0.942	1.714
CDT1 association with the CDC6:ORC:origin complex	−2.500	0.806	0.972	1.789
Dectin−1 mediated noncanonical NF−kB signaling	−2.167	0.824	1.064	1.906
NIK—>noncanonical NF−kB signaling	−2.105	0.820	1.086	1.901
Cyclin A:Cdk2−associated events at S phase entry	−2.366	0.607	1.051	2.129
SCF−beta−TrCP mediated degradation of Emi1	−2.317	0.732	1.026	2.045
Autodegradation of the E3 ubiquitin ligase COP1	−2.367	0.666	0.933	1.794
Regulation of activated PAK−2p34 by proteasome mediated degradation	−2.500	0.647	1.000	1.782
SCF(Skp2)−mediated degradation of p27/p21	−2.489	0.606	0.981	1.934

Table S3 ***TGFB2*** **gene methylation−specific Reactome pathways**

Reactome pathway	NES score *TGFB2* methylation	NES score *TGFB3* methylation	NES score *IFI27* methylation	NES score *TGFB1* methylation
STAT5 activation downstream of FLT3 ITD mutants	−1.557	−1.353	0.989	−0.724
The role of Nef in HIV−1 replication and disease pathogenesis	−1.503	−1.226	1.061	−0.950
Potential therapeutics for SARS	−1.609	−1.022	1.145	−0.859
Interleukin−2 signaling	−1.625	−1.071	1.266	−0.685
Regulation of IFNA signaling	−1.746	−1.360	1.354	−1.158
SHC−mediated cascade:FGFR1	−1.560	−1.317	0.762	1.132
Downstream signaling of activated FGFR3	−1.514	−1.294	0.801	1.169
Signaling by FGFR3 in disease	−1.568	−1.278	0.863	1.226
Signaling by FGFR3 point mutants in cancer	−1.568	−1.278	0.863	1.226
Regulation of KIT signaling	−1.635	−1.055	0.751	1.203
PECAM1 interactions	−1.628	−1.076	0.847	1.135
Signaling by NTRK3 (TRKC)	−1.718	−1.214	0.718	1.217
CD28 dependent Vav1 pathway	−1.755	−1.292	0.863	0.987
Regulation of IFNG signaling	−1.778	−1.073	0.900	1.028
Nucleotide−binding domain, leucine rich repeat containing receptor (NLR) signaling pathways	−1.864	−1.223	0.571	1.123
Signaling by FLT3 ITD and TKD mutants	−1.847	−1.359	0.832	1.358
CD28 co−stimulation	−1.688	−1.254	1.143	1.187
Erythropoietin activates Phosphoinositide−3−kinase (PI3K)	−1.525	−1.363	1.136	1.282
Inactivation of CSF3 (G−CSF) signaling	−1.540	−1.197	1.219	0.905
Signaling by CSF3 (G−CSF)	−1.531	−1.086	1.147	1.075
Interleukin−12 signaling	−1.560	−0.908	−1.239	0.745
p75NTR signals via NF−kB	−1.541	−0.828	−1.105	0.708
Interleukin−12 family signaling	−1.753	−1.096	−0.959	0.896
TAK1 activates NFkB by phosphorylation and activation of IKKs complex	−1.787	−0.864	−0.650	0.818
Sema3A PAK dependent Axon repulsion	−1.560	−1.165	−1.198	0.988
RHOH GTPase cycle	−1.510	−1.135	−1.086	1.277
SEMA3A−Plexin repulsion signaling by inhibiting Integrin adhesion	−1.492	−1.375	−1.213	−0.995

Table S4. Normal versus Tumor tissue mRNA expression in PDAC patients.

Gene	Normal Expression (Mean Log2 TPM ± SEM)	Tumor Expression (Mean Log2 TPM ± SEM)	Fold change (Tumor/Normal)	*p–value*
AURKB	−2.33 ± 0.17	2.77 ± 0.1	34.25	7.47E−215
CCNB2	−2.13 ± 0.1	2.91 ± 0.1	32.95	3.08E−210
CD28	−5.64 ± 0.17	−0.75 ± 0.13	29.75	2.83E−198
CD3D	−1.46 ± 0.14	3.16 ± 0.11	24.71	1.84E−177
CDC20	−2.27 ± 0.14	3.71 ± 0.1	62.95	5.28E−294
CDK1	−1.78 ± 0.12	3.38 ± 0.09	35.69	8.08E−220
COL11A1	−3.03 ± 0.13	4.29 ± 0.24	159.18	0.00E+00
COL1A1	3.58 ± 0.15	9.96 ± 0.13	83.48	0.00E+00
COL1A2	4.12 ± 0.15	9.93 ± 0.12	56.13	3.64E−278
COL3A1	3.62 ± 0.15	9.76 ± 0.13	70.51	4.10E−310
COL5A1	1.26 ± 0.13	6.6 ± 0.12	40.55	1.42E−235
COL5A2	0.68 ± 0.11	6.02 ± 0.12	40.61	8.95E−236
FGF5	−8.74 ± 0.16	−3.62 ± 0.2	34.86	5.71E−217
FN1	4.08 ± 0.15	9.79 ± 0.13	52.47	4.72E−269
GTSE1	−3.31 ± 0.13	1.24 ± 0.09	23.44	9.24E−172
HLA−DPA1	3.09 ± 0.14	7.47 ± 0.1	20.85	1.97E−159
HLA−DQA1	−1.59 ± 0.17	4.3 ± 0.13	59.23	0.00E+00
HLA−DQA2	−3.5 ± 0.22	2.78 ± 0.15	77.57	0.00E+00
HLA−DQB1	1.55 ± 0.15	6.16 ± 0.14	24.40	4.34E−176
HLA−DQB2	−3.65 ± 0.21	2.95 ± 0.12	97.34	0.00E+00
HLA−DRA	4.35 ± 0.15	9.3 ± 0.1	30.96	6.56E−203
HLA−DRB1	3.58 ± 0.09	7.98 ± 0.1	21.13	7.96E−161
HLA−DRB5	0.95 ± 0.18	5.84 ± 0.14	29.66	5.95E−198
IFI27	3.58 ± 0.13	9.63 ± 0.12	66.31	2.41E−301
IHH	−6.44 ± 0.21	1.85 ± 0.19	312.63	0.00E+00
IL2RA	−4.73 ± 0.18	1.23 ± 0.14	62.42	7.97E−293
LCK	−1.57 ± 0.11	3.03 ± 0.11	24.16	4.92E−175
NEK2	−5.36 ± 0.23	2.06 ± 0.11	170.36	0.00E+00
NOX4	−2.54 ± 0.14	2.75 ± 0.11	38.91	2.00E−230
NQO1	1.47 ± 0.12	7.52 ± 0.11	66.21	4.01E−301
PCDH7	−3.14 ± 0.1	2.72 ± 0.13	58.01	1.15E−282
PSMB9	1.93 ± 0.13	6.27 ± 0.08	20.26	1.66E−156
PTTG1	−0.76 ± 0.11	4.59 ± 0.08	40.62	8.44E−236
RAC2	0.79 ± 0.13	5.54 ± 0.08	26.96	4.02E−187
SEMA3A	−4.4 ± 0.13	0.47 ± 0.12	29.33	1.23E−196
SHH	−4.8 ± 0.17	1.71 ± 0.16	90.84	0.00E+00
TNC	−0.78 ± 0.12	4.07 ± 0.13	28.69	4.09E−194
TRAT1	−6.27 ± 0.22	−1.11 ± 0.16	35.60	1.68E−219
TUBB3	−0.65 ± 0.16	5.03 ± 0.09	51.19	9.65E−266
UBE2C	−2.61 ± 0.22	4.62 ± 0.11	149.96	0.00E+00
ZBP1	−3.69 ± 0.16	1.29 ± 0.13	31.58	3.12E−205
TGFB1	2.38 ± 0.13	5.42 ± 0.06	8.25	4.25E−78
TGFB2	−0.22 ± 0.12	2.76 ± 0.11	7.91	4.41E−75
TGFB3	3.83 ± 0.14	4.85 ± 0.11	2.03	3.68E−10

Table S5. Significant statistical interaction of *TGFB2* methylation and Marker Gene mRNA for genes upregulated in tumor tissues

	Gene 2 mRNA		*TGFB2* methylation		*TGFB1 methylation*		*TGFB3* methylation		*IFI27* methylation	
**Gene 2**	**HR(95% CI)**	***p*–*val***	**HR(95% CI)**	***p*–*val***	**HR(95% CI)**	***p*–*val***	**HR(95% CI)**	***p*–*val***	**HR(95% CI)**	***p*–*val***
CD3D	0.769 (0.611–0.968)	0.025	0.019 (0.002–0.178)	0.001	1.553 (1.011–2.386)	0.044	0.788 (0.507–1.223)	0.288	0.591 (0.363–0.96)	0.034
LCK	0.686 (0.506–0.93)	0.015	0.005 (0–0.11)	0.001	1.606 (1.045–2.466)	0.031	0.68 (0.442–1.045)	0.079	0.591 (0.37–0.943)	0.027
RAC2	0.667 (0.414–1.074)	0.096	0 (0–0.031)	0.003	1.61 (1.051–2.468)	0.029	0.795 (0.514–1.229)	0.301	0.58 (0.362–0.93)	0.024
HLA–DQB1	0.793 (0.635–0.991)	0.042	0.006 (0–0.181)	0.003	1.572 (1.021–2.419)	0.04	0.699 (0.453–1.081)	0.107	0.514 (0.325–0.813)	0.004
HLA–DRA	0.661 (0.461–0.949)	0.025	0 (0–0.082)	0.006	1.653 (1.074–2.546)	0.022	0.687 (0.44–1.073)	0.099	0.554 (0.349–0.877)	0.012
NOX4	0.742 (0.561–0.982)	0.037	0.015 (0.001–0.232)	0.003	1.719 (1.121–2.636)	0.013	0.628 (0.399–0.988)	0.044	0.459 (0.289–0.731)	0.001
HLA–DQB2	0.767 (0.615–0.957)	0.019	0.041 (0.005–0.336)	0.003	1.673 (1.092–2.565)	0.018	0.676 (0.435–1.051)	0.082	0.487 (0.311–0.763)	0.002
HLA–DQA1	0.85 (0.68–1.062)	0.152	0.01 (0–0.293)	0.007	1.602 (1.042–2.461)	0.032	0.74 (0.479–1.143)	0.175	0.531 (0.33–0.854)	0.009
COL3A1	0.793 (0.598–1.051)	0.106	0.001 (0–0.29)	0.017	1.737 (1.128–2.674)	0.012	0.636 (0.397–1.02)	0.06	0.466 (0.293–0.741)	0.001
COL1A1	0.811 (0.634–1.037)	0.095	0.002 (0–0.334)	0.017	1.681 (1.097–2.575)	0.017	0.624 (0.389–1)	0.05	0.468 (0.294–0.746)	0.001
HLA–DRB1	0.765 (0.557–1.05)	0.097	0.003 (0–0.403)	0.021	1.553 (1.004–2.401)	0.048	0.709 (0.457–1.098)	0.123	0.533 (0.334–0.85)	0.008
COL1A2	0.821 (0.632–1.067)	0.14	0.002 (0–0.454)	0.024	1.707 (1.112–2.62)	0.014	0.638 (0.396–1.026)	0.064	0.47 (0.296–0.748)	0.001
COL5A1	0.811 (0.612–1.075)	0.145	0.004 (0–0.469)	0.023	1.693 (1.104–2.597)	0.016	0.65 (0.405–1.043)	0.074	0.467 (0.291–0.748)	0.002
COL11A1	0.94 (0.834–1.058)	0.303	0.098 (0.017–0.583)	0.011	1.69 (1.101–2.594)	0.016	0.694 (0.445–1.083)	0.108	0.482 (0.302–0.769)	0.002

	Age at Diagnosis		Male relative to Female		*TGFB2* meth × Gene 2 interaction		Univariate Gene 2 mRNA	
**Gene 2**	**HR(95% CI)**	***p*–*val***	**HR(95% CI)**	***p*–*val***	**HR(95% CI)**	***p*–*val***	**HR(95% CI)**	***p*–*val***
CD3D	1.03 (1.009–1.052)	0.005	0.811 (0.532–1.236)	0.33	1.693 (1.197–2.395)	0.003	1.025 (0.904–1.161)	0.705
LCK	1.028 (1.006–1.05)	0.012	0.805 (0.528–1.226)	0.312	1.774 (1.218–2.584)	0.003	1.102 (0.957–1.269)	0.178
RAC2	1.026 (1.005–1.048)	0.015	0.816 (0.536–1.242)	0.343	2.528 (1.333–4.792)	0.004	1.296 (1.06–1.586)	0.012
HLA–DQB1	1.03 (1.009–1.051)	0.005	0.921 (0.606–1.4)	0.701	1.487 (1.104–2.003)	0.009	1.077 (0.953–1.216)	0.236
HLA–DRA	1.032 (1.01–1.054)	0.004	0.839 (0.549–1.281)	0.416	1.767 (1.143–2.732)	0.01	1.129 (0.959–1.329)	0.146
NOX4	1.028 (1.006–1.05)	0.011	0.879 (0.58–1.334)	0.545	1.58 (1.117–2.235)	0.01	1.188 (1.028–1.373)	0.02
HLA–DQB2	1.032 (1.01–1.054)	0.004	0.811 (0.528–1.247)	0.34	1.42 (1.066–1.89)	0.016	1.03 (0.91–1.167)	0.636
HLA–DQA1	1.031 (1.01–1.053)	0.004	0.959 (0.628–1.465)	0.847	1.46 (1.064–2.004)	0.019	1.097 (0.968–1.243)	0.146
COL3A1	1.029 (1.007–1.05)	0.008	0.86 (0.566–1.305)	0.478	1.449 (1.039–2.022)	0.029	1.196 (1.039–1.378)	0.013
COL1A1	1.028 (1.007–1.05)	0.01	0.842 (0.555–1.278)	0.419	1.386 (1.028–1.869)	0.032	1.166 (1.022–1.329)	0.022
HLA–DRB1	1.031 (1.01–1.053)	0.004	0.875 (0.577–1.328)	0.53	1.528 (1.023–2.28)	0.038	1.137 (0.973–1.328)	0.105
COL1A2	1.028 (1.007–1.05)	0.009	0.853 (0.562–1.294)	0.455	1.397 (1.011–1.93)	0.043	1.2 (1.038–1.387)	0.014
COL5A1	1.028 (1.006–1.049)	0.011	0.857 (0.565–1.3)	0.468	1.42 (1.011–1.995)	0.043	1.215 (1.042–1.417)	0.013
COL11A1	1.028 (1.006–1.05)	0.011	0.882 (0.582–1.335)	0.552	1.174 (1.001–1.377)	0.048	1.115 (1.032–1.204)	0.006
